# Primary extraosseous dural chondrosarcoma: a case report

**DOI:** 10.1186/s12883-021-02515-y

**Published:** 2021-12-15

**Authors:** Francis Garay Buitron, Albert Pons-Escoda, Noemí Vidal, Alberto Torres, Angels Camins

**Affiliations:** 1 Radiology Department, Hospital Universitari de Bellvitge, Barcelona, Spain; 2 Pathology Department, Hospital Universitari de Bellvitge, Barcelona, Spain; 3 Neurosurgery Department, Hospital Universitari de Bellvitge, Barcelona, Spain

**Keywords:** Chondrosarcoma, Dura mater, Meningeal neoplasms, Diagnostic imaging, Case report

## Abstract

**Background:**

Dural chondrosarcoma is a very rare intracranial tumor, given that meninges do not normally contain cartilaginous tissue from which it can originate. We present a case of primary extraosseous dural chondrosarcoma.

**Case presentation:**

A 48-year-old woman presented to our tertiary center neurosurgery consultation with progressive headache, vomiting, vertigo, and gait instability of 5 months’ duration.

An initial brain CT revealed a large parietal mass with gross calcifications and subtle hyperostosis of the inner table. Subsequent brain MRI showed a heterogeneous expansive lesion with a honey-comb enhancement.

Discussion of intra- or extra-axial location was warranted, and finally, initial presurgical suspicion of meningioma arose although some atypical imaging features were detected. The differential diagnosis included solitary fibrous tumor–hemangiopericytoma and dural metastasis.

Total resection of the lesion was performed, extra-axial origin was confirmed, and pathology resulted in a primary dural chondrosarcoma.

**Conclusion:**

The importance of this case presentation lies in the unusual nature of the final diagnosis, the brief literature review and differential diagnosis with emphasis on imaging pearls, as well as the useful reminder for physicians to consider less frequent diseases when key findings do not unambiguously lead to the usual suspects.

## Background

Chondrosarcoma is a malignant tumor characterized by the formation of a cartilaginous matrix. It is usually located in the appendicular skeleton; however, in few cases it is found inside the skull and even more rare it has a meningeal origin where cartilaginous tissue is usually absent [1, 2]. We present a case of intracranial dural chondrosarcoma and its differential diagnosis with emphasis on imaging pearls.

## Case presentation

We report the case of a 48-year-old woman who presented to neurosurgery consultation with a five-month history of progressive headache, vomiting, vertigo, and gait instability. Neurologic examination also revealed a subtle bradypsychia.

**Fig. 1:**
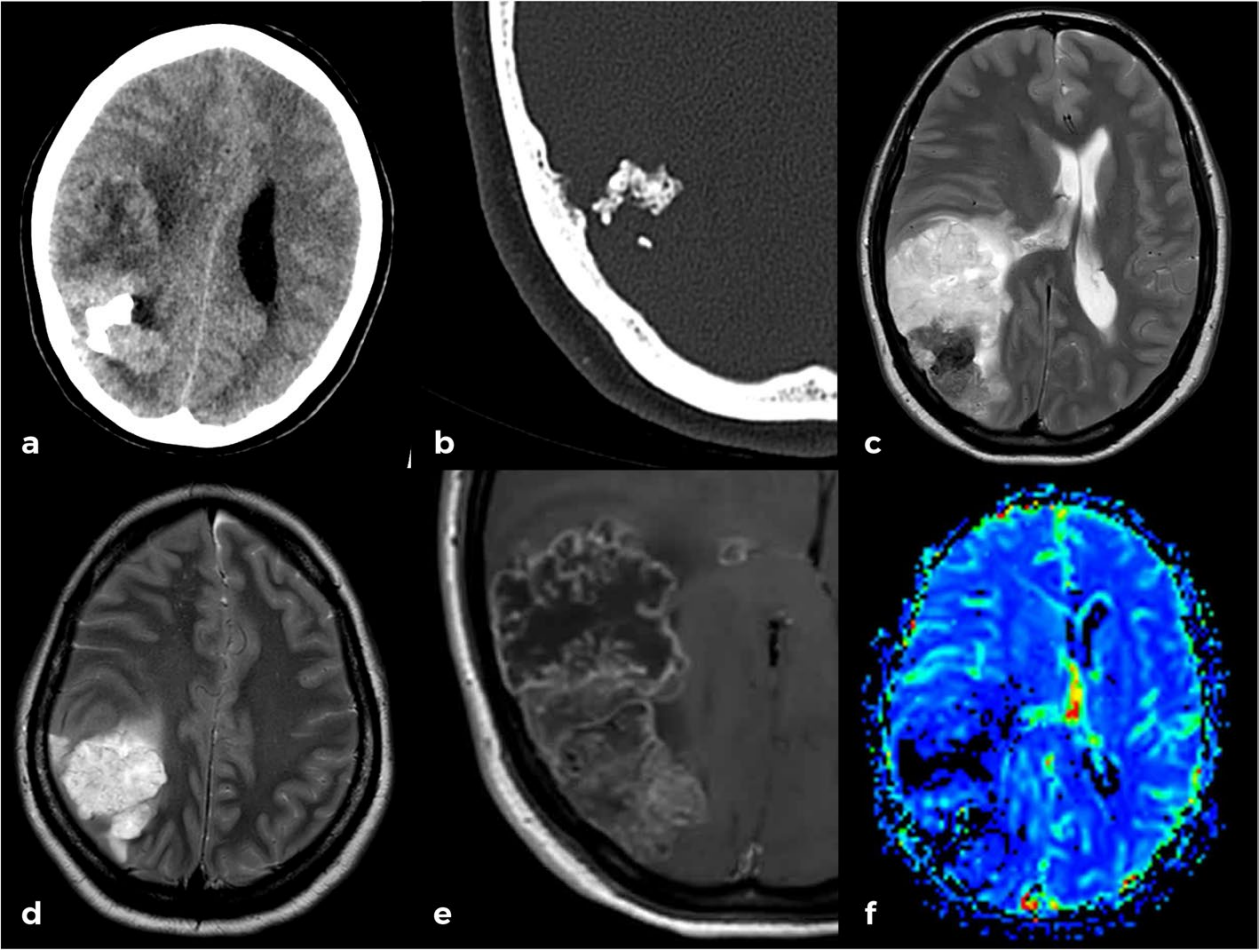
Axial brain-parenchyma (**a**) and bone windows (**b**) of brain in non-enhanced CT demonstrating a parietal mass with subtle hyperostosis and internal calcifications. Axial T2 WI (**c,d**) shows a lobulated mass which is predominantly hyperintense but with some foci of low signal. Significant mass effect and subtle edema is noted. T1-WI postcontrast (**e**) and axial T2* MRI perfusion (**f**) images show a heterogeneous enhancement with honeycomb pattern and low CBV of the tumor

Non-enhanced CT (Philips Incisive CT) showed a large right parietal mass with gross calcifications. Serious doubts about intra- or extra-axial origin of the lesion arose. Nevertheless, the detection of a subtle underlying hyperostotic spicule on the inner table revealed the extra-axial nature of the lesion (Fig. 1a, b).

Brain MRI (Philips Ingenia 1.5 T) confirmed an expansive-lobulated mass in the right parietal region with mild peripheral edema. It was hypointense on T1-weighted images (WI) and hyperintense with foci of low signal intensity on T2-WI. A partial CSF-cleft and heterogeneous gadolinium enhancement with an internal honey-comb pattern could also be assessed. It showed a strikingly low cerebral blood volume (CBV) on dynamic susceptibility contrast- perfusion-weighted imaging (DSC-PWI). There were no signs of diffusion restriction. With an extra-axial location in mind, spectroscopy did not uncover any helpful specific biomarkers such as presence of alanine for meningioma, high myo-inositol for solitary fibrous tumor-hemangiopericytoma (SFT-HPC), or high mobile lipids for metastasis (Fig. 1c-f).

**Fig. 2:**
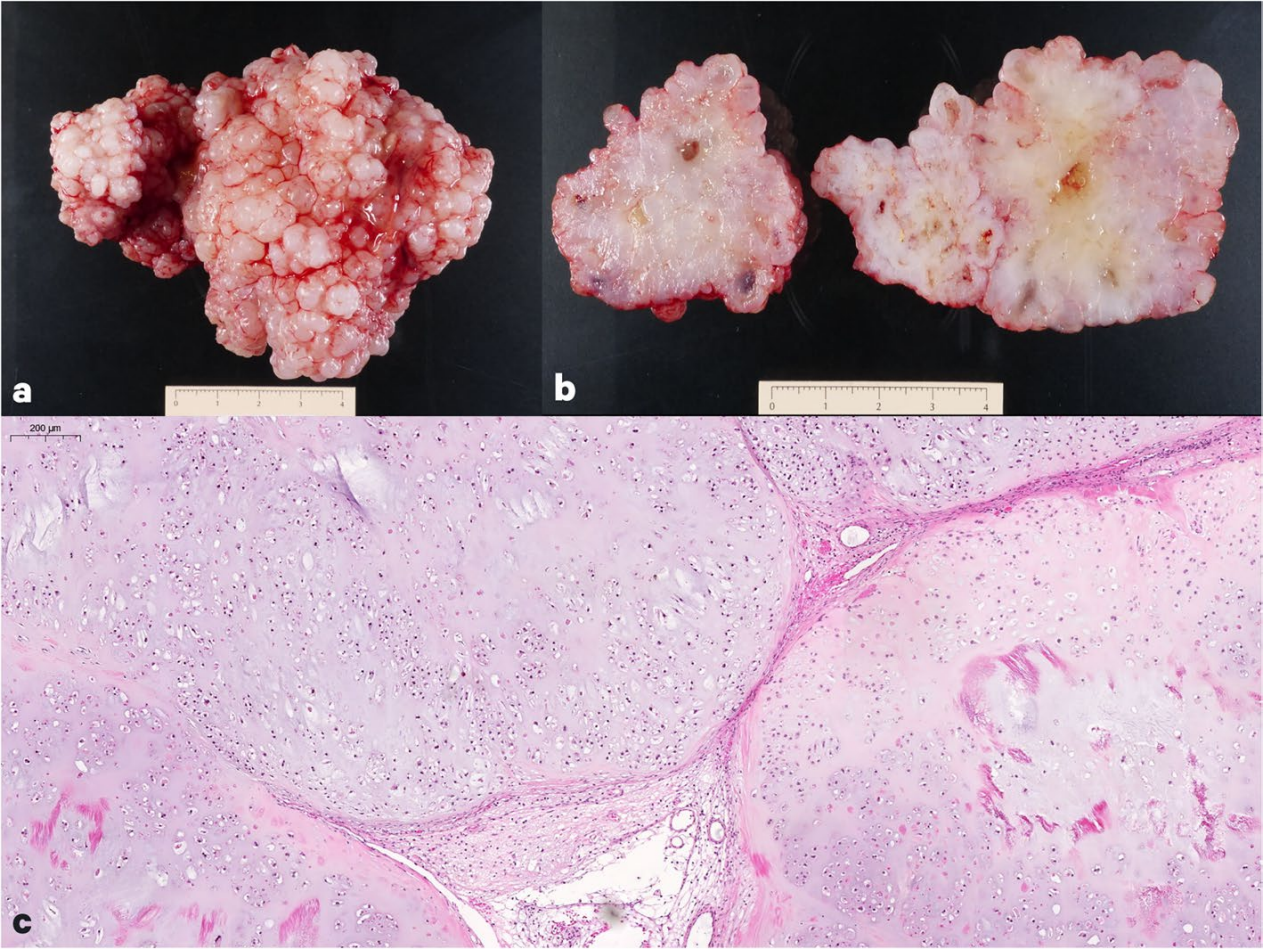
Polylobulated mass with glistening nodules, focal softening, and liquefaction (**a,b**). Moderate cellular tumor with abundant chondroid matrix. Chondrocytes showed low-to-moderate atypia, binucleated cells, and necrosis (**c**)

Total neurosurgical resection of the tumor was performed, confirming an extra-axial origin. Histology demonstrated a moderate degree of cellularity with chondroid matrix, chondrocytes of low-to-moderate atypia, binucleated forms, and tumor necrosis (Fig. 2a-c). Pathology led to the final diagnosis of conventional dural chondrosarcoma grade II, a malignant entity in the 2020 WHO classification of chondrogenic bone tumors [1].

Finally, negative whole-body positron-emission-tomography-CT confirmed the extraosseous primary origin.

The patient was treated with adjuvant radiotherapy following surgery. Clinical follow-up revealed an incomplete right hemianopsia as a sequala. The first follow-up MRI 2 months after surgery showed no signs of tumor recurrence.

## Discussion and conclusion

In the present case, we were faced with a middle-aged woman with an intracranial lesion showing wide dural base, internal calcifications, hyperostotic spicules on underlying inner table of the skull, partial CSF-cleft, and honey-comb enhancement. With this semiology, meningioma was considered the most probable diagnosis, but low CBV and the absence of meningioma-specific biomarkers on spectroscopy suggested differential diagnosis was in order. The absence of myo-inositol went against SFT-HPC while the absence of a known primary malignancy, the unifocality of the tumor, and the absence of an aggressive underlying bone pattern did not support metastasis. With all these considerations in mind, the preoperative radiological diagnosis was of meningioma, considering SFT-HPC and dural metastasis as less probable differentials [3–7].

Meningioma is the most frequent intracranial and dural tumor in adults. It predominates in middle-aged women. The most common imaging features are dural tail sign, avid enhancement, and the presence of a CSF-cleft between the lesion border and brain cortex. Intratumoral calcifications and hyperostosis are imaging pearls, with the latter being highly specific. Nevertheless, meningiomas are very ubiquitous and polymorphic tumors, which warrant challenging differential diagnosis with other less frequent extra-axial lesions that can share some imaging features. In this scenario, advanced imaging sequences play an important role in the presurgical diagnosis. Extremely high CBV and low PSR values on DSC-PWI and the presence of alanine on spectroscopy can be diagnostic clues for meningioma [3–5].

Isolated dural metastases are uncommon, even though a known history of neoplasm may raise this suspicion. The two most common manifestations are multifocal nodular masses and diffuse thickening of the dura. This may be associated with aggressive lytic, permeative, or blastic bone patterns and strong enhancement. The dural tail may be present, but calcifications are rare. On spectroscopy, there are high lipid values in relation to necrosis; DSC-PWI reveals elevated CBV and decreased PSR, but to a lesser degree than in meningioma [3, 4, 6].

Amongst mesenchymal tumors, SFT-HPC is the most frequent in this location. It is more frequent in middle-aged men, with an earlier age than patients with meningioma. It presents as a lobulated mass, usually with a focal dural attachment instead of a wide dural base. There is no associated hyperostosis, while underlying focal bone lysis is frequent. Calcifications are very uncommon. SFT-HPC may be mushroom shaped, and it may show flow voids. It may enhance heterogeneously due to a greater tendency than meningioma to present necrosis, cystic degeneration, or hemorrhage. On advanced MRI sequences, the key finding is a high myo-inositol peak on spectroscopy [3–5, 7].

However, in the present case, the pathology study ruled out these presurgical diagnoses, and the lesion was labelled as chondrosarcoma. It is the third most common primary malignant bone tumor (20–27%). It predominates in adults after 40 years of age and is slightly more frequent in males [1, 8]. It usually affects the appendicular skeleton. Intracranial chondrosarcoma is very rare, representing 0.16% of all brain tumors. Most of these arise in the skull base, developing from espheno-petrous and petro-clival synchondrosis that contain cartilage. Exceptionally, and based on isolated case reports, they can arise in convexity meninges; but the dura does not normally contain cartilage, so few hypotheses have been proposed about their origin like the presence of abnormal embryonal rests of cartilaginous tissue or multipotential mesenchymal cells in the meninges [2, 8, 9].

Histologically, three types of intracranial and dural chondrosarcoma have been reported: conventional (grade I-III), mesenchymal, and myxoid. Classic or conventional chondrosarcomas usually are avascular and have few or no mesenchymal components and no myxoid stroma. In contrast, the mesenchymal subtype is the most malignant, highly vascular, and often associated with metastasis [2, 10].

Some authors have described the radiological features of dural chondrosarcoma. This lesion is usually a lobulated dural tumor with low enhancement, intratumoral calcifications, a very hyperintense signal on T2WI, and islands of low signal intensity due to well-differentiated cartilage. Other features include the absence of a dural tail and mild vasogenic edema. The presence of honeycomb enhancement and poor vascularity is characteristic of classic chondrosarcoma, and these are clearly depicted in our case [2, 10].

Radical surgical resection is the treatment of choice, and adjuvant radiotherapy is often considered, especially after incomplete resection [8, 9]. Therefore, the relevance of an appropriate pre-surgical diagnosis orientation could be very important when facing a dural mass as it may directly impact patient management. Indeed, some meningiomas can be managed with follow-up strategies, dural metastases can sometimes be treated with systemic oncologic treatments; while malignant mesenchymal tumors such as our case may directly go through a maximal safe resection and probably posterior adjuvant radiotherapy [8, 9, 11, 12].

A precise bibliographic review on intracranial dural chondrosarcomas above the skull base is hindered because they are often treated altogether with skull-base (different origin in synchondrosis cartilage) and most reports are from some decades ago with incomplete and non-comparable data with nowadays. Nonetheless, the scarce comparable literature collects similar characteristics to those of the current case and some assumptions can be extracted: 1. the disease frequently involves young and middle-aged patients with a possible slightly male predilection; 2. radiology hallmarks are chondroid calcifications, T2-hyperintensity, honey-comb enhancement and signs of poor vascularity; 3. surgical resection and adjuvant radiotherapy is the accepted treatment of choice; and 4. overall prognosis is good in general terms due to an often-indolent growth, rare metastases and the implementation of adequate treatments [2, 8–10].

The importance of this case presentation lays on the rarity of the presented diagnostic with its fully available clinical information. And also, on the always-useful reminder for physician to consider less frequent diseases when any of the imaging pearls do not convincingly lead to the usual suspects, because an accurate pre-surgical diagnostic may directly impact patients’ management.

## Data Availability

Not applicable.

## Abbreviations

CT: Computed tomography
MRI: Magnetic resonance imaging
WI: Weighted images
CBV: Cerebral blood volume
DSC-PWI: Dynamic susceptibility contrast- perfusion-weighted imaging
SFT-HPC: Solitary fibrous tumor-hemangiopericytoma
WHO: World health organization
PSR: Percentage of signal intensity recovery
